# Acromioclavicular joint reconstruction implants have differing ability to restore horizontal and vertical plane stability

**DOI:** 10.1007/s00167-021-06700-x

**Published:** 2021-08-26

**Authors:** Mohamed Alkoheji, Hadi El-Daou, Jillian Lee, Adrian Carlos, Livio Di Mascio, Andrew A. Amis

**Affiliations:** 1 Department of Orthopaedics, Royal London Hospital, Whitechapel Road, London, E1 1FR UK; 2 Biomechanics Group, Mechanical Engineering Department, Imperial College London, London, SW7 2AZ UK

**Keywords:** Acromioclavicular joint reconstruction, Artificial implants, Biomechanics, Robotic testing, Vertical and horizontal plane stability

## Abstract

**Purpose:**

Persistent acromioclavicular joint (ACJ) instability following high grade injuries causes significant symptoms. The importance of horizontal plane stability is increasingly recognised. There is little evidence of the ability of current implant methods to restore native ACJ stability in the vertical and horizontal planes. The purpose of this work was to measure the ability of three implant reconstructions to restore native ACJ stability.

**Methods:**

Three groups of nine fresh-frozen shoulders each were mounted into a robotic testing system. The scapula was stationary and the robot displaced the clavicle to measure native anterior, posterior, superior and inferior (A, P, S, I) stability at 50 N force. The ACJ capsule, conoid and trapezoid ligaments were transected and the ACJ was reconstructed using one of three commercially available systems. Two systems (tape loop + screw and tape loop + button) wrapped a tape around the clavicle and coracoid, the third system (sutures + buttons) passed directly through tunnels in the clavicle and coracoid. The stabilities were remeasured. The data for A, P, S, I stability and ranges of A–P and S–I stability were analyzed by ANOVA and repeated-measures Student *t* tests with Bonferroni correction, to contrast each reconstruction stability versus the native ACJ data for that set of nine specimens, and examined contrasts among the reconstructions.

**Results:**

All three reconstructions restored the range of A–P stability to that of the native ACJ. However, the coracoid loop devices shifted the clavicle anteriorly. For S–I stability, only the sutures + buttons reconstruction did not differ significantly from native ligament restraint.

**Conclusions:**

Only the sutures + buttons reconstruction, that passed directly through tunnels in the clavicle and coracoid, restored all stability measures (A, P, S, I) to the native values, while the tape implants wrapped around the bones anteriorised the clavicle. These findings show differing abilities among reconstructions to restore native stability in horizontal and vertical planes. (300 words)

## Introduction

Acromioclavicular joint (ACJ) injuries constitute 9% of shoulder girdle injuries [3]. The classic Rockwood classification correlates poorly with clinical symptoms, primarily due to its inability to accurately predict the anatomical structures injured in each case [7]. Clinical evaluation of ACJ stability has been based on anterior–posterior (AP) radiographs to measure superior displacement of the lateral clavicle [18], while it is difficult to measure horizontal displacement [1]. This is why methods of ACJ stabilisation have traditionally focused on preventing superior displacement of the clavicle [15, 20].

Many methods of ACJ stabilisation have been described, with no clear gold standard [1, 13, 17, 21]. Recently a more three dimensional understanding of the ACJ has developed, with a better appreciation of the restraints of horizontal displacements [8, 12, 22] and of the ligamentous structures [19]. Scheibel et al. [23] recognised persistent symptomatic horizontal plane instability in a high number of reconstructions that focused on coraco-clavicular (CC) ligament reconstruction alone. Whatever method is used for ACJ reconstruction it must be able to reproduce as closely as possible the native joint stability in both the vertical and horizontal planes. Jari et al. [9] measured superior and AP stability of the native and reconstructed ACJ using a robot. However, despite the availability of that test method, there has been little data published on the stability of ACJ reconstructions in both planes.

Several manufacturers have introduced artificial implants for ACJ stabilisation, with variations among their configurations of tapes and sutures and their fixation means, but there is little data available on their ability to stabilise the ACJ. Therefore a biomechanical study has been conducted to compare the potential of three commonly used reconstruction techniques based on artificial implants to restore the native stability (laxity) of the superior suspensory complex of the shoulder. This information should be clinically useful in guiding the choice and use of implants for ACJ reconstruction.

The aim of this work was to determine whether the three reconstruction methods could reproduce native ACJ stability in both vertical and horizontal planes. The null hypothesis was that there would not be significant differences among the three reconstruction methods, nor versus the native ACJ stability.

## Materials and methods

### Specimen preparation

Following Imperial College Healthcare Tissue Bank Research Ethics Committee approval R18045, 31 fresh-frozen cadaveric shoulders were obtained. Four were used in a pilot study, leaving 27 for analysis: 12 male and 15 female aged 55 ± 13 years. Each specimen comprised the scapula, clavicle, and proximal half-length of the humerus, with intact skin and soft tissues. Specimens were thawed at room temperature for 24 h prior to preparation.

The skin and overlying soft tissues were excised to expose the ACJ capsular ligaments, coracoid, clavicle, and trapezoid and conoid ligaments. The humeral shaft, scapular body and clavicle were exposed. The scapula inferior to the posterior spine, and the medial clavicle, were cleared of soft tissues. Twenty-five mm of the inferior corner of the scapula, and 20 mm of the medial clavicle were excised. The prepared medial end of the clavicle was secured in a cylindrical metal pot with polymethylmethacrylate bone cement. The inferior part of the scapular body was similarly secured in a rectangular metal mounting.

### Robotic stability testing

The scapula mounting was fixed rigidly to the base of a robotic joint testing system (TX90, Staubli AG, Horgen, Switzerland). This system included a load cell which could measure in 6 degrees of freedom (DoF) (Omega 85, ATI Industrial Automation Co., Apex, NC). Previous work had validated this system to  ± 0.11 mm in translation and  ± 0.13**°** in rotation and in both native and prosthetic human joints [2, 14] using the method of Fujie [5]. The load cell was mounted onto the free end of the robot arm and the clavicle secured to it via a tubular mounting [12]. The ‘neutral’ datum point was established by moving the clavicle to minimise the forces and torques across the ACJ. The robot was initially operated in ‘force control’ to measure motion of the clavicle when a displacing force was imposed in a chosen direction. As the robot moved, it monitored any coupled forces that built up in other DoF and moved to minimise them. This led to a path of motion being defined while maintaining a constant rotational orientation. Force and displacement data were recorded continuously.

To measure the stability of the intact ACJ complex, the clavicle was moved in the AP and SI directions at a speed of less than 50 mm/min until a displacing force of 50 N was reached. The AP movement was in the horizontal plane and SI movement vertical. The orientation of the AP axis was defined by taking the coronal plane to bisect the angle between the plane of the scapula and long axis of the clavicle when the shoulder was viewed from the superior aspect. The pilot study on four shoulders confirmed that repeated displacements to the motion limits reached at 50 N displacing force with the ACJ intact did not cause irreversible elongation of the soft-tissues stabilising the ACJ; a similar study had used 70 N [9]. All tests were run three times in A, P, S and I directions.

The robot was switched off and the conoid and trapezoid ligaments and the ACJ capsule were transected with the clavicle held at the anatomical (neutral load) position. The specimens were separated into three groups of nine, which each received one of three reconstruction methods.

When the robot was switched on after the ACJ reconstruction, there was usually a load acting on the clavicle, due to tension in the reconstruction. The robot was then moved to a new neutral position. This movement was recorded, so that all data were related to the original position of the native ACJ. The robot then repeated the stability tests as before, moving the clavicle in 3 DoF of translation but without coupled rotations until the 50 N limit was reached. The displacement (mm) away from the neutral position was recorded. This was done three times for each specimen in A, P, S and I directions.

In all subsequent results, the stabilities presented are movement of the clavicle in relation to the fixed scapula.

### Reconstruction methods

#### Tape loop plus screw fixation reconstruction

**Fig. 1:**
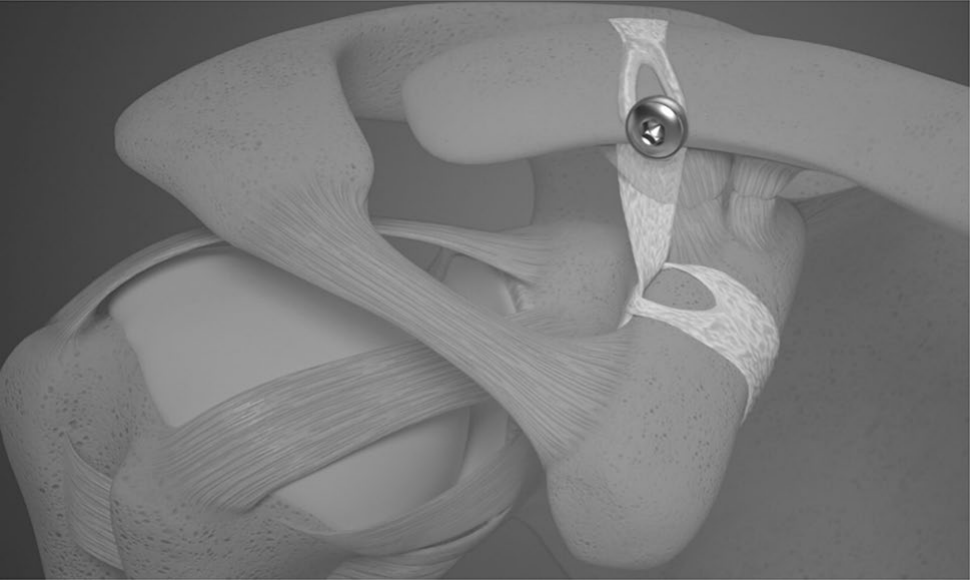
Tape loop + screw fixation reconstruction ( ^©^ Reproduced with permission of Lockdown Surgical Inc., Chanhassen, MN, USA)

The Tape loop + screw fixation implant (Lockdown, Lockdown Surgical Inc., Chanhassen, MN, USA) was used according to the manufacturer’s recommended technique (Fig. 1). The conoid tubercle was identified and provisionally marked for more accurate placement. The lateral end of the clavicle was not excised as reduction was achievable without impingement in all cases. The base of the coracoid was exposed both medially and laterally to allow a hooked passing instrument (Lockdown Surgical Inc.) to be passed under it from medial to lateral. Once the tip was visualised, the length gauge was passed through. The metal tip of the length gauge was then passed through a loop in the gauge, to cinch it around the base at the apex of the ‘knuckle’ of the coracoid. The metal tip was then passed behind the clavicle at the level of the conoid tubercle and pulled over from posterior to anterior. A marking on the length gauge was read on the anterior cortex of the clavicle to determine the required implant length. The length gauge was passed back under the clavicle and uncinched, then used to pass the implant around the base of the coracoid and back over the clavicle in the described technique. Using the length gauge to assist in holding the reduction, a 2.5 mm AP tunnel was drilled, exiting lateral to the synthetic ligament on the posterior aspect of the clavicle. The length of the hole was measured and a 3.5 mm cortical bone screw was passed through the hard loop of the ligament and into the drilled hole to secure the ligament.

#### Tape loop plus button fixation reconstruction

**Fig. 2:**
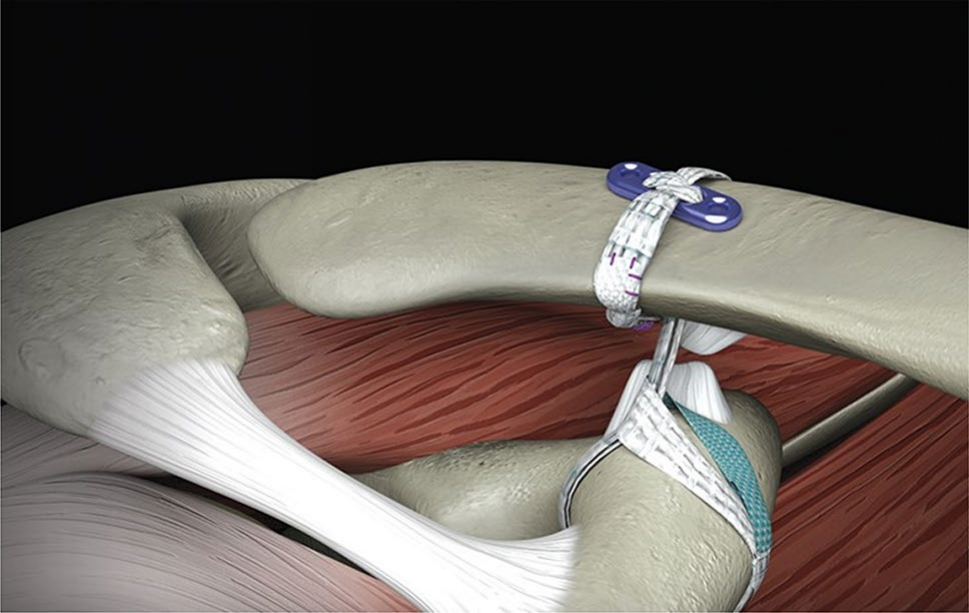
Standard Tape loop + button fixation reconstruction. ( ^©^ Reproduced with permission of Xiros Ltd., Leeds, UK)

The manufacturer’s technique for the Tape loop + button fixation implant (Infinity-Lock, Xiros Ltd., Leeds, UK) (Fig. 2) exposed the coracoid and a side-specific disposable hooked passing instrument (Xiros Ltd.) was passed underneath the coracoid from medial to lateral. A nitinol loop was passed through the hook and grasped a suture on the looped end of the synthetic ligament tape to pull it under the coracoid. The free ends of the implant were passed through the loop and the implant was cinched to the base of the coracoid, ensuring that it was at the apex of the ‘knuckle’ of the coracoid. A 4-mm hole was drilled through the clavicle at the level of the conoid tubercle, in the centre of the width of the clavicle and aiming at the base of the coracoid, using a guidewire and cannulated drill bit. The nitinol loop was passed through the hole to retrieve the free ends of the implant from below the clavicle. The ends of the implant were passed through a titanium alloy button and a single throw was made over it. One free end was passed over the posterior aspect of the clavicle and retrieved from underneath it, then the two ends were tied in a knot at the anterior aspect of the clavicle, avoiding a large bulk on top of the button (Fig. 2). Optional locking sutures in the knot were not placed during the biomechanical study.

#### Transosseous sutures with buttons reconstruction

The manufacturer’s standard technique was used for the transosseous sutures + buttons reconstruction (FiberTape sutures with Dog Bone buttons, Arthrex GmbH, Naples, FL, USA).

**Fig. 3:**
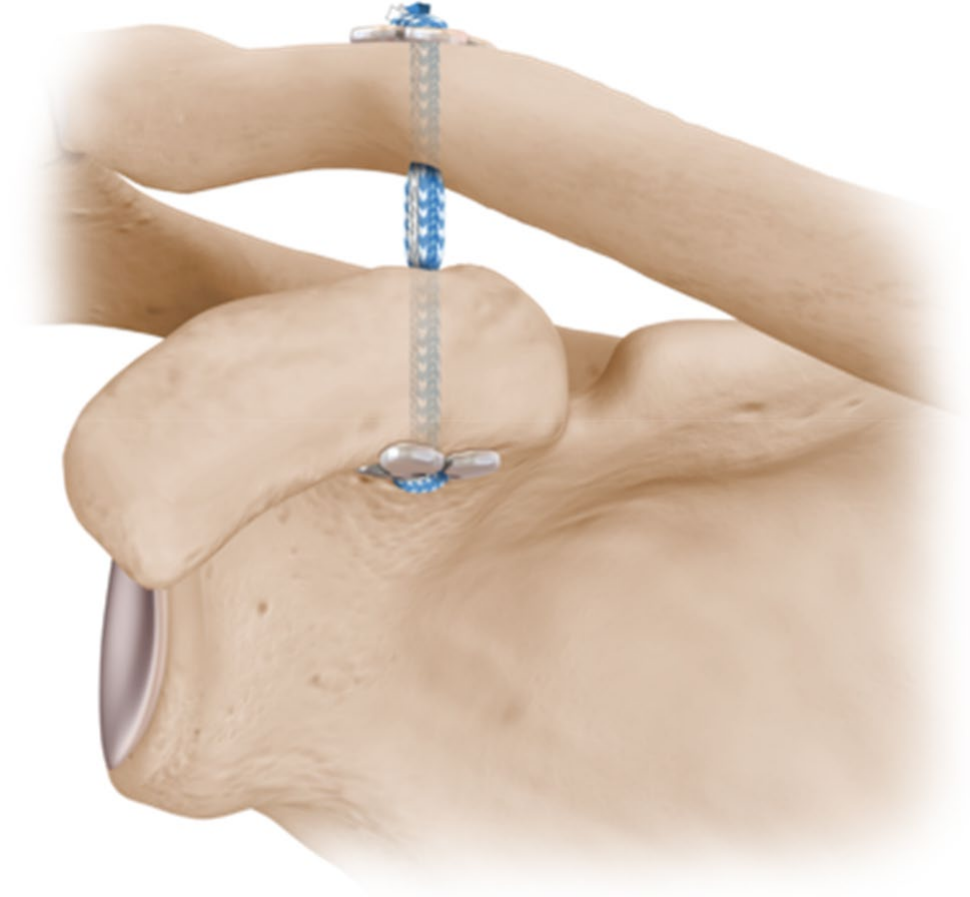
Transosseous sutures + buttons reconstruction. ( ^©^ Reproduced with permission of Arthrex GmbH, Naples, FL, USA)

The reconstruction used two 3-mm drill holes, one at the base of the coracoid and another through the clavicle directly above it, positioned colinearly with the ACJ reduced. Two sutures with an attached titanium alloy button were shuttled up through the drill holes and then passed through another titanium alloy button above the clavicle (Fig. 3). The button below the coracoid was checked to ensure that it was snug to the bone prior to tightening the sutures and tying them in a knot on top of the button on the clavicle.

### Statistical analysis

A power analysis based on published data [9] using G*Power v 3.1.9.7 showed that a change of laxity of 3 mm could be identified with 95% confidence and 82% power with eight specimens per test group.

The Shapiro–Wilk test confirmed that data were distributed normally with *p* = 0.05. For each of the three reconstruction methods the data were analyzed to calculate the change of stability from the native to the reconstructed state, with nine shoulders per group, in each test direction: A, P, S, I. Increased stability meant that the ACJ was less lax—overconstrained—post reconstruction, and vice-versa.

The ability of the three reconstructions to restore native stability was examined in each loading direction by one-way analysis of variance, with differences taken to be significant with *p* < 0.05. The data for each of the three reconstructions were examined for changes of stability from the native by two-way repeated-measures Student *t* tests, with *p* < 0.0167 with Bonferroni correction. The changes of stability from native values among the three reconstruction methods were then contrasted by unpaired two-way *t* tests, with *p* < 0.0167 with Bonferroni correction.

## Results

### Anterior stability

**Fig. 4:**
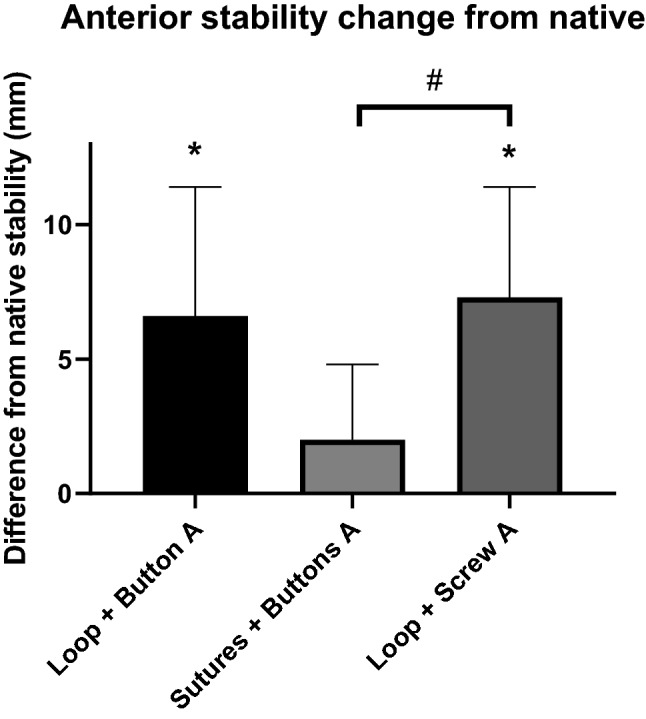
Change of anterior (A) stability (mm) from the native joint stability for each of the tape loop plus button, transosseous sutures plus buttons and tape loop plus screw reconstructions. * significant difference from native stability (*p* < 0.0167), # significant difference between reconstructions (*p* = 0.0052). (mean + SD, *n* = 9 per group)

The ability of the three methods to restore native anterior stability differed significantly (*p* = 0.0180, Fig. 4). Post-testing found a significant difference of residual instability between the sutures plus buttons and tape loop plus screw methods (*p* = 0.0052). Both of the tape loop reconstruction methods that wrapped around the clavicle (with screw or button fixation) allowed residual anterior instability to persist (*p* < 0.0167), while the trans-osseous sutures method did not.

### Posterior stability

**Fig. 5:**
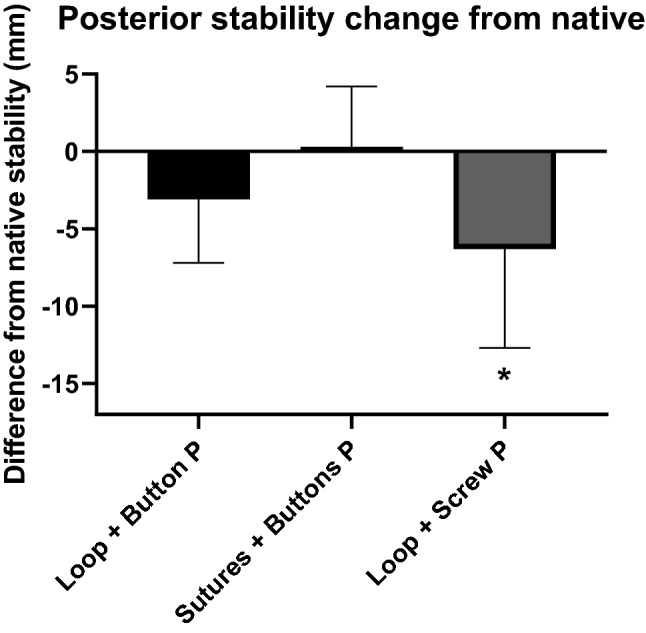
Change of posterior (P) stability (mm) from the native joint for each of the tape loop plus button, transosseous sutures plus buttons and tape loop plus screw reconstructions. * significant difference from native stability (*p* < 0.001). (mean + SD, *n* = 9 per group)

The ability of the three reconstruction methods to restore native posterior stability differed significantly (*p* = 0.0312, Fig. 5). Although the tape loop plus screw fixation reconstruction caused overconstraint (*p* < 0.001), post-testing did not find significant differences among the reconstructions (n.s.).

### Range of AP stability

The tape loop plus button procedure gave a range of AP stability (laxity) that did not differ significantly from the native ACJ. The anterior stability was significantly under-constrained, posterior stability tended to be overconstrained, so the mean AP position of the clavicle shifted 5 mm anteriorly.

The transosseous sutures plus buttons procedure gave range of AP stability, anterior stability and posterior stability that did not differ significantly from the native ACJ. The non-significant tendencies to underconstrain anteriorly and overconstrain posteriorly shifted the mean AP position 2 mm anteriorly.

The tape loop plus screw procedure overconstrained posterior displacement of the clavicle, and underconstrained anterior displacement. Although the range of AP stability was not found to differ significantly from the native stability, the mean AP position of the clavicle was shifted 7 mm anteriorly.

### Superior stability

**Fig. 6:**
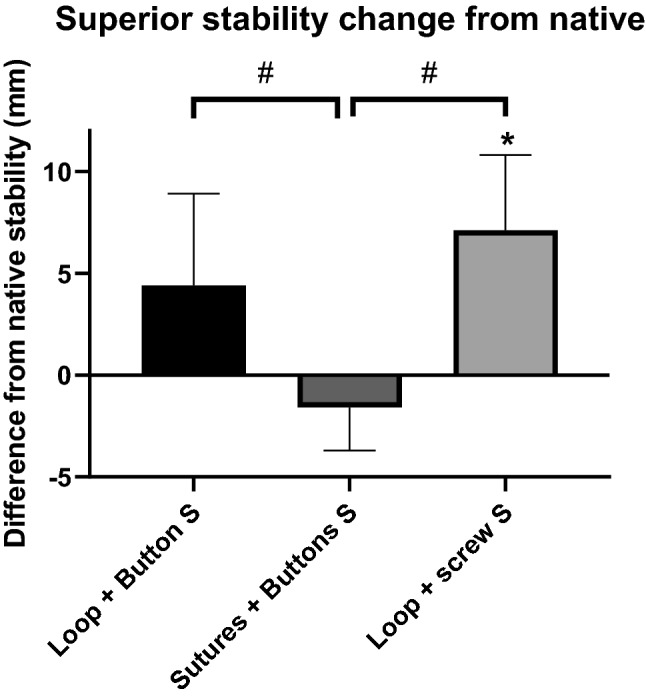
Change of superior (S) stability (mm) from the native joint for each of the tape loop plus button, transosseous sutures plus buttons and tape loop plus screw reconstructions. * significant difference from native stability, # significant difference between reconstructions. (mean + SD, *n* = 9 per group)

The superior stability of the three reconstruction methods differed significantly (*p* < 0.001, Fig. 6). The transosseous sutures plus buttons method restored native stability closer than the tape loop plus screw (*p* < 0.001) and the tape loop plus button (*p* = 0.0023) methods. The tape loop plus screw reconstruction allowed residual superior instability to persist (*p* = 0.0004) while the tape loop plus button did not (n.s.).

### Inferior stability

**Fig. 7:**
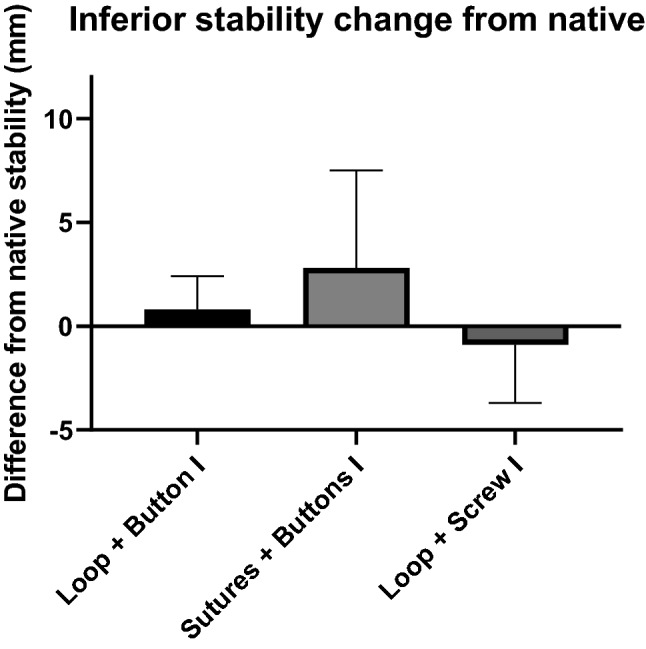
Change of inferior (I) stability (mm) from the native joint for each of the tape loop plus button, transosseous sutures plus buttons and tape loop plus screw reconstructions. (mean + SD, *n* = 9 per group)

The ability of the three reconstruction methods to restore native inferior stability did not differ significantly (n.s., Fig. 7). The reconstructions did not leave persisting residual instability (n.s.).

### Range of superior-inferior stability

The tape loop plus button procedure was significantly less stable in S-I than the native ACJ (*p* = 0.0035). However, each of the superior and inferior stabilities was not significantly different to native stability (n.s.). These combined effects shifted the mean position of the clavicle 2 mm superiorly.

The transosseous sutures plus buttons procedure gave range of SI, superior, and inferior stabilities that did not differ significantly from the native ACJ. The tendencies to over-constrain superiorly and under-constrain inferiorly shifted the mean SI position of the clavicle inferiorly by 2 mm.

The tape loop plus screw procedure under-constrained superior displacement of the clavicle, and the inferior stability matched the native ACJ, so the reconstructed ACJ was significantly less stable in S-I than the native ACJ (*p* = 0.0014). The mean position of the range of SI displacements was elevated 4 mm after this reconstruction.

## Discussion

The main finding of this study was that the three ACJ stabilisation methods had differing abilities to restore native ACJ stability in SI and AP directions, thus the null hypothesis was rejected. All three methods restored AP stability to native values, but the tapes which wrapped around the coracoid displaced the clavicle anteriorly from its native resting position, with resulting anterior instability and posterior overconstraint. The anterior and posterior stabilities and the range of AP stability were restored to native values by the transosseous sutures plus buttons construct. The native range of SI stability was restored by the transosseous sutures plus buttons construct, but the other two constructs left significant underconstraint. The superior laxity of the reconstructed ACJ did not differ significantly from the native laxity for the transosseous sutures plus buttons and tape loop plus button constructs.

Few biomechanical studies have compared implants for treatment of ACJ injuries despite their increasing availability. Ladermann et al. [11] compared ACJ and CC cerclage sutures with a suture tape plus bone anchors construct and a superior clavicle hook plate [24], finding that the cerclage sutures mimicked physiological behaviour more closely. Tulner et al. [25] reported that the tape loop plus screw fixation (Lockdown Surgical Inc) implant shifted the clavicle anteriorly, as in the present study, but this had no clinical importance. The superior displacement was similar to controls immediately post operatively but increased 4 mm by 6 months post-reconstruction. The present study found that this implant tended to overconstrain posterior displacement of the clavicle in relation to the acromion. This may be clinically beneficial if it resists anterior displacement of the scapula and collapse under load in vivo, but the reduced anterior/superior restraint when tested in the robot is a clinical surgical consideration when undertaking these procedures.

Reconstructions that require tunnels through the coracoid have led to fractures due to malposition or excessive sizing of the tunnels or possible attritional wear from polyethylene core suture material [3, 6, 16]. Fracture of the base of the coracoid has also been reported [10]. Techniques in the present study minimise coracoid fracture risk by requiring only 3 or 4 mm tunnels. There were no fractures of the coracoid, but this work was not a study of load to failure.

The transosseous sutures reconstruction gave the closest return to the native SI stability, with significant differences for the coracoid loop devices. This might be because it is difficult to remove all the excess slack and soft tissue interposition below the coracoid. In comparison, the trans-coracoid device is easier to tighten because the sutures pass directly between the two titanium buttons.

Clinical studies of the three implants have indicated similar patient satisfaction and complication rates. This should be taken into consideration along with the results of this biomechanical study when selecting an implant for this procedure.

The reconstructions were performed by four fellowship trained upper limb surgeons using the manufacturers’ techniques. However, the placement of drill holes and tensioning of knots was left to each surgeon’s individual judgment and were influenced by the anatomy of each specimen. This reflects real life circumstances during the use of these implants by different surgeons.

A limitation of the experiment was that it only measured stability at ‘time zero’, as though intra-operatively, and could not account for longer-term biological or mechanical effects which may allow return of laxity post-surgery [4]. The robot held the clavicle at its native position while the reconstructions were performed, after the soft tissues had been transected. This consistent test configuration avoided the need to remove/replace the specimen at each stage of surgery. A strength of this experimental design is the repeated-measures analysis of the reconstruction stability versus the native stability for each joint.

This work may be useful for the clinician in demonstrating the ability of the artificial constructs to restore native ACJ stability in both the vertical and horizontal planes. This study supports the use of the three commonly used synthetic AC joint ligament repair methods. The coracoid looped reconstructions in vitro have been shown to lead to anterior instability and posterior overconstraint. This may explain some previously reported anterior displacement of the clavicle in vivo which has not been shown to cause any clinical sequelae, and indeed may be beneficial in resisting physiological loads [25].

## Conclusion

All three implants restored the native range of horizontal plane stability of the ACJ, but the tapes that looped around the coracoid shifted the resting position of the lateral clavicle anteriorly. The transosseous coracoid suture device also provided better superior stability than the tape loop devices, with the closest reproduction of the native ACJ stability.
